# “Designer cytokines” targeting the tumor vasculature—think global and act local

**DOI:** 10.15252/emmm.201911801

**Published:** 2020-01-09

**Authors:** Thomas Kammertoens, Josephine Kemna, Matthias Leisegang

**Affiliations:** ^1^ Institute of Immunology Charité—Universitätsmedizin Berlin, Campus Buch Berlin Germany; ^2^ Max‐Delbrück‐Center for Molecular Medicine Berlin Germany

**Keywords:** Cancer, Chemical Biology, Immunology

## Abstract

Tumor necrosis factor (TNF) was discovered in 1975 as a lipopolysaccharide‐induced serum factor that causes necrosis of tumors (Carswell *et al*, 1975). It was later found that TNF and cachectin, a factor causing wasting disease, were one and the same molecule (Beutler *et al*, 1985). Studies on the inflammatory activity of TNF have been translated into clinical success, namely blocking antibodies used to suppress autoimmune diseases. Research on TNF anti‐tumor activity, in contrast, has not yet resulted in a therapeutic breakthrough. This may change, based on a study by Huyghe *et al* (2020) describing novel “designer cytokines” (TNF and interferon‐γ) that increase local activity by targeting the CD13‐positive tumor vasculature, while simultaneously lowering the binding affinity to the respective cytokine receptor, thereby reducing off‐target effects on normal cells.

## Graphical abstract

Despite TNF anti‐tumor activity, its therapeutic use has been limited by high toxicity. T. Kammertoens and colleagues discuss the work from Huygue *et al*, in this issue of *EMBO Molecular Medicine,* which describes new cytokines engineered to overcome these limitations.

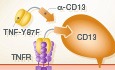

Tumor necrosis factor (TNF) and interferon‐γ (IFNγ) are essential to reject solid tumors and act both on tumor and stromal cells. In fact, the anti‐tumor effects of TNF and IFNγ on activated tumor vasculature are probably the reason why immunotherapy using T cells can destroy solid tumors that are refractory to cytotoxic therapies (Kammertoens *et al*, 2017). Because anti‐tumor effects of TNF and IFNγ had been observed in pre‐clinical models, both cytokines were tested in the clinic when recombinant proteins became available. However, severe systemic toxicities limited their clinical use (Fig 1A). The therapeutic potential of high doses of TNF and IFNγ in humans could only be demonstrated upon shielding of the organism from systemic toxicity via separation of the circulation of a cancer‐affected limb from the rest of the body, a procedure called “isolated limb perfusion” (ten Hagen *et al*, 2008).

**Figure 1:**
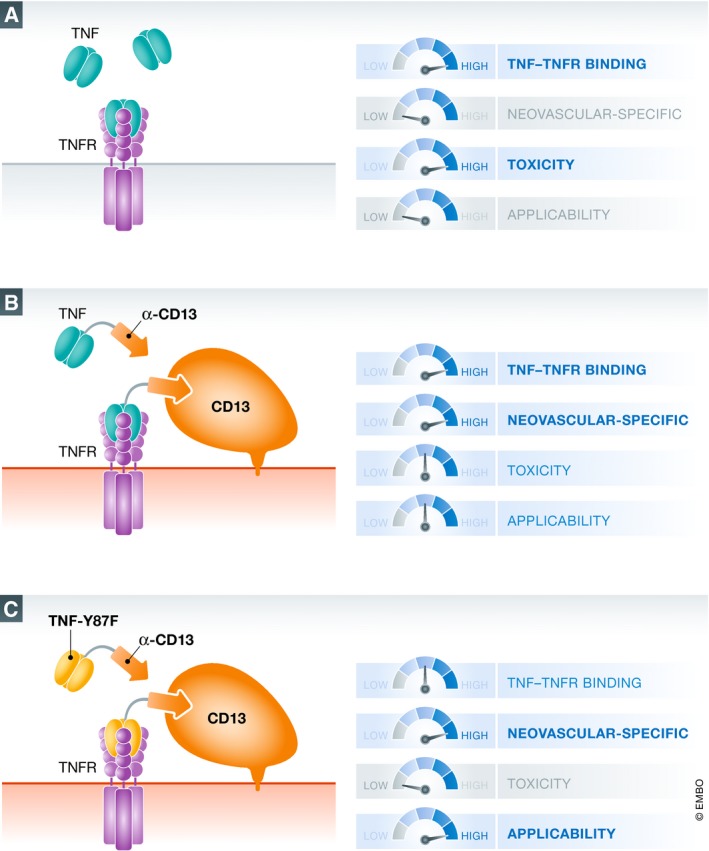
Strategies to use TNF as an anti‐cancer agent (A) Using recombinant tumor necrosis factor (TNF) that binds to the TNF receptor (TNFR) on all cells in the body causes toxicity and prohibits clinical application. (B) Fusing TNF to a single‐chain antibody targeting CD13 that is preferentially expressed on tumor vasculature concentrates its activity to the tumor site but does not exclude off‐target effects as it still binds to TNFR on all other cells in the body. (C) The novel “designer cytokine” approach developed by Hyghe *et al* combines selective targeting of TNF to the tumor vasculature using a CD13‐specific single‐chain antibody and mutating the TNF molecule (Y87F) to decrease the binding to TNFR and avoid systemic toxicity.

Two different strategies have been developed to reduce systemic toxicity of TNF or IFNγ while preserving their anti‐tumor activity. The first one consists in specifically targeting the cytokines to CD13, a marker predominantly expressed on tumor vasculature. Targeting was achieved using either peptides or single‐chain antibodies directed against CD13 (Fig 1B). Pre‐clinical data suggest that such approaches are feasible and may improve local therapeutic anti‐tumor activity (Johansson *et al*, 2012; Corti *et al*, 2013). The second strategy to reduce unwanted side effects is based on changing the molecular interactions between TNF or IFNγ and their receptors. A prime example of such a strategy is the study by Mendoza *et al* (2019) in which, based on the crystal structure of the IFNγ signaling complex, specific signaling agonists were designed by changing contact residues between the ligand and receptor chains, resulting in a molecule with reduced side effects for immunotherapy.

In their study, Huyghe *et al* (2020) have combined both strategies and thus improved efficacy and safety for the therapeutic use of TNF and IFNγ (Huyghe *et al*, 2020). First, they succeeded in increasing the local cytokine concentration at the tumor site by attaching a CD13‐specific single‐chain antibody to the cytokines. Second, they mutated TNF (by changing amino acid 87 Y to F) and IFNγ (by truncating 8 C‐terminal amino acids), thereby reducing the biological activity approx. 10,000‐ and 7,000‐fold, respectively. This resulted in decreased systemic toxicity (Fig 1C). The novel TNF could also improve adoptive T‐cell therapy using T cells engineered with chimeric antigen receptors by increasing the number of T cells infiltrating the tumor. In mice with endothelial‐specific TNFR1 expression, tumors could be eradicated without measurable toxicity. However, tumor‐activated vessels are not the only ones highly susceptible to the destructive TNF effects, also vessels exposed to bacterial products react strongly to TNF with systemic or local Schwarzman reactions (Rothstein & Schreiber, 1988). It would therefore be important to test whether endothelial cells that are activated during bacterial infections could become CD13‐positive targets of the novel designer cytokines. Finally, it should be mentioned that there is a third powerful strategy to locally release therapeutic amounts of TNF and IFNγ, which is the transfer of tumor antigen‐specific T cells that release the cytokines upon antigen encounter.

Clinical studies that specifically direct cytokines such as TNF or IFNγ to the tumor endothelium via peptides or single‐chain antibodies are already underway. Huyghe *et al*'s elegant approach, however, makes it possible to concentrate the effect of the cytokines more precisely to the tumor site, thereby increasing the therapeutic effect and avoiding negative side effects and systemic toxicity. Therefore, this work is certainly an important step forward for the development of “designer cytokines” that are suitable for clinical application.
